# The total xanthones extracted from *Gentianella acuta* alleviates HFpEF by activating the IRE1α/Xbp1s pathway

**DOI:** 10.1111/jcmm.18466

**Published:** 2024-06-07

**Authors:** Linna Zhao, Yiping Qin, Yangong Liu, Liping An, Weizhe Liu, Chuang Zhang, Qiuhang Song, Cheng Dai, Juanjuan Zhang, Aiying Li

**Affiliations:** ^1^ Hebei Key Laboratory of Chinese Medicine Research on Cardio‐Cerebrovascular Disease Shijiazhuang Hebei China; ^2^ The First Hospital of Hebei Medical University Shijiazhuang Hebei China; ^3^ College of Basic Medicine Hebei University of Chinese Medicine Shijiazhuang Hebei China; ^4^ Department of Technology Hebei University of Chinese Medicine Shijiazhuang Hebei China; ^5^ Faculty of Nursing Hebei University of Chinese Medicine Shijiazhuang Hebei China

**Keywords:** cardiac remodelling, cardiomyocyte apoptosis, heart failure with preserved ejection fraction, IRE1α/Xbp1s signalling pathway, total xanthones extracted from *Gentianella acuta*

## Abstract

Heart failure with preserved ejection fraction (HFpEF) is a clinical syndrome characterized by pulmonary and systemic congestion resulting from left ventricular diastolic dysfunction and increased filling pressure. Currently, however, there is no evidence on effective pharmacotherapy for HFpEF. In this study, we aimed to investigate the therapeutic effect of total xanthones extracted from *Gentianella acuta* (TXG) on HFpEF by establishing an high‐fat diet (HFD) + L‐NAME‐induced mouse model. Echocardiography was employed to assess the impact of TXG on the cardiac function in HFpEF mice. Haematoxylin and eosin staining, wheat germ agglutinin staining, and Masson's trichrome staining were utilized to observe the histopathological changes following TXG treatment. The results demonstrated that TXG alleviated HFpEF by reducing the expressions of genes associated with myocardial hypertrophy, fibrosis and apoptosis. Furthermore, TXG improved cardiomyocyte apoptosis by inhibiting the expression of apoptosis‐related proteins. Mechanistic investigations revealed that TXG could activate the inositol‐requiring enzyme 1α (IRE1α)/X‐box‐binding protein 1 (Xbp1s) signalling pathway, but the knockdown of IRE1α using the IRE1α inhibitor STF083010 or siRNA‐IRE1α impaired the ability of TXG to ameliorate cardiac remodelling in HFpEF models. In conclusion, TXG alleviates myocardial hypertrophy, fibrosis and apoptosis through the activation of the IRE1α/Xbp1s signalling pathway, suggesting its potential beneficial effects on HFpEF patients.

## 1 INTRODUCTION

Heart failure with preserved ejection fraction (HFpEF) is a clinical syndrome characterized by symptoms and signs of heart failure in the presence of normal or near‐normal left ventricular ejection fraction (LVEF). Typically, an LVEF of 50% or higher is considered within the normal range.^1^ There are diverse clinical manifestations of HFpEF, encompassing dyspnoea, fatigue and oedema. These symptoms primarily arise from elevated left ventricular (LV) filling pressure due to ventricular diastolic dysfunction, resulting in pulmonary and systemic congestion.^1^

The relatively intricate pathogenesis of HFpEF is associated with a multitude of pathophysiological processes. At the cardiac level, primary alterations involve cardiomyocyte diastolic dysfunction, cardiac structure modifications (such as myocardial hypertrophy and fibrosis) and cardiac inflammation. At the systemic level, potential changes may include systemic inflammatory state, endothelial dysfunction, obesity, anaemia and renal insufficiency.^2^ Current studies on the molecular mechanisms of HFpEF primarily focus on cardiac remodelling, cardiac inflammation and oxidative stress.^3^, ^4^, ^5^ The elucidation of these mechanisms is conducive to better understanding the pathophysiological process of HFpEF and providing new prevention and treatment strategies. Previous studies have shown that inositol‐requiring enzyme 1α (IRE1α) plays a key role in the pathogenesis and progression of HFpEF.^6^, ^7^ IRE1α is one of the most conservative signalling pathways implicated in the endoplasmic reticulum stress response, which serves as an important player in cellular stress response. It will be activated in the case of endoplasmic reticulum stress. Activated IRE1α can splice X‐box binding‐protein 1 (Xbp1) mRNA, leading to the production of active Xbp1 protein and regulation of a variety of stress response genes.^8^ Regarding HFpEF, cardiomyocytes may be subjected to various stresses, resulting in continuous activation of endoplasmic reticulum stress response and then triggering cardiomyocyte apoptosis. Therefore, it has the potential to inhibit cardiomyocyte apoptosis by modulating the IRE1α signalling pathway, thereby preventing the development of HFpEF.

Traditional Chinese medicine ingredients have shown favourable cardiovascular protective effects with minimal side effects, becoming suitable for long‐term use and effective in treating cardiovascular diseases. *Gentianella acuta*, a bitter and cool‐tasting annual herb belonging to the Gentianaceae family, is extensively employed in Ewenki folk medicine and Mongolian medicine to address such conditions as arrhythmia and coronary heart disease, exhibiting significant therapeutic efficacy. Previous studies have indicated that *G. acuta* can inhibit myocardial fibrosis and enhance cardiac function.^9^ Moreover, xanthones have been identified as the major active components responsible for inhibiting myocardial fibrosis, alleviating myocardial hypertrophy and improving cardiac function.^9^, ^10^, ^11^ However, it remains unclear whether traditional Chinese herbal medicine for treating HFpEF can effectively ameliorate the complex cardiac condition characterized by unique pathogenesis mechanisms. In this study, we tried to investigate the therapeutic effect of total xanthones extracted from *G. acuta* (TXG) on HFpEF by establishing an HFD + L‐NAME‐induced HFpEF mouse model. The results revealed that TXG successfully ameliorated HFpEF‐related organ changes in mice while alleviating diastolic dysfunction. Furthermore, it attenuated pulmonary oedema along with increase in LV wall thickness caused by HFpEF‐induced LV remodelling. These findings suggest that TXG may serve as a natural and efficacious protective agent against HFpEF. Mechanistic investigations further unveiled that TXG effectively suppressed HFpEF‐induced myocardial hypertrophy, fibrosis and apoptosis through activating the IRE1α/Xbp1 signalling pathway. Our results suggested that TXG provides novel strategies for relieving HFpEF.

## 2 MATERIALS AND METHODS

### 2.1 Plant material and standard for TXG

*G. acuta* sourced from Hulunbeier, Inner Mongolia and was authenticated by experts at the University. Standards for demethylbellidifolin, bellidifolin, swertianolin and mangiferin were acquired from Chinese companies. Norswertianolin was synthesized in the research laboratory. These steps were designed to ensure the authenticity and quality of the plant material and establish reliable reference standards for the chemical components of interest, which are crucial for accurate research on medicinal plants.

The extract of Pseudogentiana acuminata was prepared by means of methanol reflux extraction and silica gel column chromatography. The chromatography was performed on a Waters XBridge C18 (250 mm × 4.6 mm, 5 μm) column with gradient elution of acetonitrile‐0.1% phosphoric acid solution at a volume flow rate of 1.0 mL/min and column temperature of 35°C. The detection wavelength was 254 nm, and the components of mangiferin, norswertianolin, swertianolin, demethylbellidifolin and bellidifolin were identified (Figure S1).

### 2.2 Ethics statement and animals

C57BL/6N mice (8‐week old) were purchased from Beijing Vital River Laboratory Animal Technology Co. Ltd., China (Licence No. SCXK 2016‐0006). The mice were raised under standard temperature (23 ± 1°C), humidity (40%–70%) and light–dark conditions (light/dark ratio: 12/12 h), with free access to food and water, for 7 days before the experiment. The Institutional Animal Care and Use Committee issued references for all the animal experimental procedures and approval for the study protocol (DWLL2021090).

Forty C57BL/6N mice were randomly divided into four groups: Control group, Control + TXG group, HFpEF group and HFpEF + TXG group (*n* = 10 per group). All mice were administered 0.1 mL/10 g of TXG liquid or water daily via gavage. The mice in the Control group and HFpEF group were regularly administered with water. In the Control + TXG group and HFpEF + TXG group, the mice were regularly administered with water containing TXG (4 mg/kg/day). As for the mice in HFpEF group, high‐fat diet with 60% fat was provided for specified periods of time, along with N[w]‐nitro‐l‐arginine methyl ester (L‐NAME; 0.5 g/L, Sigma Aldrich) added to the drinking water after adjusting the pH to 7.4.^6^, ^7^, ^12^, ^13^ Regarding HFpEF + TXG group, the mice were fed with high‐fat diet with the addition of L‐NAME (0.5 g/L) to the drinking water, along with the addition of TXG (4 mg/kg/day). Tissue samples from animals, including the heart and lungs, were collected post‐euthanasia, gas anaesthesia using isoflurane to the mice prior to euthanasia, followed by decapitation to minimize their suffering.

### 2.3 Echocardiography analysis

The VEVO 2100 system (Visual Sonics, Toronto, ON, Canada) was utilized to perform echocardiography for evaluating LV systolic function under isoflurane‐induced anaesthesia. Standard LV examination encompassed measurements of LV end‐diastolic posterior wall thickness (LVPWd), LV end‐diastolic internal diameter (LVEDd) and LV end‐diastolic anterior wall thickness (LVAWd). Pulsed‐wave Doppler parameters were employed to assess LV diastolic function, including mitral inflow peak early filling velocity (*E*) and velocity at atrial contraction (*A*), followed by calculation of the (*E/A*) ratio. All echocardiogram data were analysed using Vevo Lab 3.2.0 software.

### 2.4 Histopathological analysis

The excised cardiac tissue was promptly rinsed with saline and subsequently fixed at room temperature for 24 h in a 10% neutral buffered formalin solution. For the evaluation of histological abnormalities and collagen deposition, the sections were stained using haematoxylin and eosin as well as Masson's trichrome. Finally, images were captured by an optical microscope (Leica DM4000B, Leica Microsystems GmbH, Wetzlar, Germany).

### 2.5 Wheat germ agglutinin (WGA) staining

The paraffin sections underwent conventional dewaxing, heat‐mediated antigen retrieval and cooling at room temperature. Subsequently, the sections were incubated with WGA staining solution at 37°C for 30 min. The sections were washed three time with phosphate‐buffered saline (PBS), air‐dried and then treated with 4′’,6‐diamidino‐2‐phenylindole (DAPI) staining solution away from light. After incubation at room temperature for 10 min, the slices were air‐dried and sealed with an anti‐fading agent. Finally, the images were visualized and captured under a fluorescence microscope, followed by quantitative analysis of the cell area using the ImageJ software.

### 2.6 Cell culture

The H9c2 cell line (rat cardiomyocytes) was procured from Procell (Wuhan, China). The cells were cultured in Dulbecco's modified Eagle medium (DMEM, Gibco, USA) supplemented with 10% fetal bovine serum (BI, Israel), penicillin (100 units/mL) and streptomycin (100 μg/mL), and maintained at 37°C in a 5% CO_2_ atmosphere. In the intervention experiment with STF083010 (an IRE1α inhibitor), H9c2 cells were first treated with angiotensin II (AngII) (1 μM) and DMSO‐dissolved STF083010 (100 μM) (Catalogue #HY‐15845, MedChemExpress). After this initial treatment, the cells were exposed to 50 μM TXG for 48 h. To suppress the expression of IRE1α in H9c2 cells, the cells were first transfected with the specific small interference RNA (siRNA) targeting IRE1α. Following transfection, the cells were either treated with 50 μM TXG or left untreated for 48 h.

### 2.7 Cell transfection

The siRNAs were designed and synthesized by GenePharma (Shanghai, China) (siRNA: 5′‐CCUUUCUCCCAGAUCCUAATT‐3′, 5′‐UUAGGAUCUGGGAGAAAGGTT‐3′). Lipofectamine™ 3000 transfection kit (Invitrogen) was used for siRNA transfection. At 48 h post‐transfection, cells were harvested for subsequent experiments.

### 2.8 Quantitative real‐time polymerase chain reaction (qRT‐PCR) analysis

Total RNA was extracted from the heart tissue using the Total RNA kit II (R6934‐01, Omega, USA), and cDNA was synthesized using the MonScript™ RTIII All‐in‐one Mix in conjunction with a dsDNase kit (MR05101M, Monad, China). QRT‐PCR analysis was performed using the QuantStudio 1 real‐time PCR instrument (Thermo Fisher Scientific, USA). The expression levels of IRE1α, Xbp1s and c‐MYC were quantified using the MonAmp™ ChemoHS qPCR Mix kit (MQ00401S, Monad, China), with glyceraldehyde‐3‐phosphate dehydrogenase (GAPDH) mRNA as the internal control for target mRNA quantification. The primers were synthesized by Shenggong Bioengineering (Shanghai, China) (Table 1).

**TABLE 1:** The specific sequences of the primers for qRT‐PCR.

Gene	Primer	Sequence (5′–3′)
GAPDH	Forward	AGGTCGGTGTGAACGGATTTG
	Reverse	TGTAGACCATGTAGTTGAGGTCA
IRE1α	Forward	AACAACCTGCCCAAACATCG
	Reverse	GAGATACGGTGGTCGGTGTG
XBP1s	Forward	GAGTCCGCAGCAGGTG
	Reverse	GTGTCAGAGTCCATGGGA
c‐MYC	Forward	ATGCCCCTCAACGTGAACTTC
	Reverse	CGCAACATAGGATGGAGAGCA

### 2.9 Western blotting analysis

Heart tissue and H9c2 cells were lysed with RIPA lysis buffer containing phenylmethylsulfonyl fluoride (PMSF), protease inhibitor cocktail (Roche, Switzerland) and phosphatase inhibitors (Wuhan Servicebio, China) to extract proteins. The protein concentration was measured using the BCA protein quantification kit (PC0020, Solarbio, China). Totally 30 μg of protein was separated by SDS‐PAGE, then transferred to a PVDF membrane (Millipore, USA) and incubated with primary antibodies. The dilution of each antibody is as follows: anti‐IRE1α (rabbit polyclonal, 1:1000, DF7709, Affinity), anti‐Phospho‐IRE1α (Ser724) (rabbit polyclonal, 1:1000, #AF7150, Affinity), anti‐Xbp1s (rabbit polyclonal, 1:1000, 24868‐1‐AP, Proteintech), anti‐c‐MYC (mouse polyclonal, 1:5000, 67447‐1‐Ig, Proteintech), anti‐B‐cell lymphoma‐2 (Bcl‐2) (mouse polyclonal, 1:2000, 68103‐1‐Ig, Proteintech), anti‐Cleaved caspase 3 (Asp175) (rabbit polyclonal, 1:1000, #9661, Cell Signalling Technology), anti‐Bcl‐2‐associated X protein (Bax) (rabbit polyclonal, 1:1000, GB11690, Servicebio), anti‐NPPA (rabbit polyclonal, 1:2000, 27426‐1‐AP, Proteintech), anti‐brain natriuretic peptide (BNP) (mouse polyclonal, 1:1000, ab239510, Abcam), anti‐beta‐myosin heavy chain (β‐MHC) (rabbit polyclonal, 1:1000, 22280‐1‐AP, Proteintech), anti‐Collagen I (rabbit polyclonal, 1:1000, 14695‐1‐AP, Proteintech), anti‐Collagen III (rabbit polyclonal, 1:1000, 22734‐1‐AP, Proteintech), anti‐alpha‐smooth muscle actin (α‐SMA) (rabbit polyclonal, 1:4000, ab32575, Abcam) and anti‐GAPDH (mouse monoclonal, 1:10,000, 60004‐1‐Ig, Proteintech). The immunoreactive bands were detected using the ECL Chemiluminescent Substrate Kit and visualized using the chemiluminescent imager OmegaLum W (Minneapolis, MN, USA).

### 2.10 Terminal deoxynucleotidyl transferase‐mediated dUTP nick end labelling (TUNEL) assay

The heart tissue obtained from the mice in each experimental group was fixed with 4% paraformaldehyde, followed by paraffin embedding and sectioning at a thickness of 5 μm. After dewaxing and hydration, the myocardial tissue sections were stained using a TUNEL staining kit (PF0006, Proteintech) and then examined under the fluorescence microscope. Excitation was performed at the wavelength of 450–500 nm, while emission was implemented at the wavelength of 515–565 nm. The TUNEL positive rate in the myocardial tissue was calculated accordingly.

### 2.11 Recording of tail cuff blood pressure

The blood pressure of the mice in the four experimental groups was monitored using a small animal blood pressure meter (BP‐2000). The mice were placed in a temperature‐controlled dark chamber at 37°C with their tails exposed. After an initial acclimation period, a pressure sensor was placed on the upper third of each mouse's tail. Once a stable sine wave pattern appeared on the screen, the pressure sensor was activated to capture measurements and record data. This process was repeated three times for each animal to obtain the mean value.

### 2.12 Exercise exhaustion test

In order to assess the endurance capacity, the mice in the four experimental groups received exercise training sessions lasting for 3 days on a motorized treadmill (model: DB030 experimental treadmill) under controlled climatic conditions. After that, all mice underwent an exhaustive running test on the same DB030 treadmill at a constant incline of 20° uphill gradient. The initial phase involved warming up at a speed of 5 m/min for 4 min before increasing the speed to 14 m/min for two additional minutes. Subsequently, the speed was incremented at 2 m/min every 2 min until the point of exhaustion, which was defined as the failure to resume running within 10s following direct contact with the electrical stimulation grid. The running time and distance were recorded and then calculated.

### 2.13 Immunohistochemical staining

Immunohistochemical staining was conducted to detect the protein expressions of Collagen I, Collagen III and α‐SMA according to the instructions of the PV‐9000 kit (OriGene, Delaware USA). The heart tissue samples were made into 5‐μm‐thick sections following paraffin immersion. Next, the sections were dewaxed with xylene, rehydrated in an ethanol gradient and treated with 0.01 mol/L citric acid buffer (pH = 6.0) for 15 min of antigen retrieval at 100°C. Afterward, the sections were incubated in 3% H_2_O_2_ to reduce endogenous peroxidase activity and blocked with 10% goat serum for 30 min. Next, the tissue samples were incubated with anti‐Collagen I (1:500, rabbit polyclonal, 14695‐1‐AP, Proteintech), anti‐Collagen III (1:500, rabbit polyclonal, 22734‐1‐AP, Proteintech) and anti‐α‐SMA (1:500, rabbit polyclonal, ab32575, Abcam) primary antibodies at 4°C overnight. Following incubation with secondary antibodies for 30 min at room temperature, the sections were rinsed with PBS and treated with DAB for colour development. Finally, images were captured under the optical microscope (Leica DM4000B, Leica Microsystems GmbH, Wetzlar, Germany).

### 2.14 Enzyme‐linked immunosorbent assay (ELISA)

The serum obtained through centrifugation at 1000 rpm for 15 min was stored at −20°C prior to testing. AngII level in the serum was measured using a commercial ELISA kit (Shanghai Senxiong Technology Industry Co., Ltd., China) according to the manufacturer's instructions, with OD values measured at 450 nm using a microplate reader.

### 2.15 Statistical data analysis

The experimental data (mean ± standard deviation [SD]) were analysed using one‐way analysis of variance, followed by Tukey's multiple comparisons test via SPSS 22.0 software (IBM, Chicago, IL, USA). Statistical significance was determined at the threshold of *p* < 0.05. GraphPad Prism 9.0 software (GraphPad Software, Inc., USA) was utilized for chart plotting.

## 3 RESULTS

### 3.1 TXG ameliorated cardiac function in HFD + L‐NAME‐induced HFpEF mice

**FIGURE 1:**
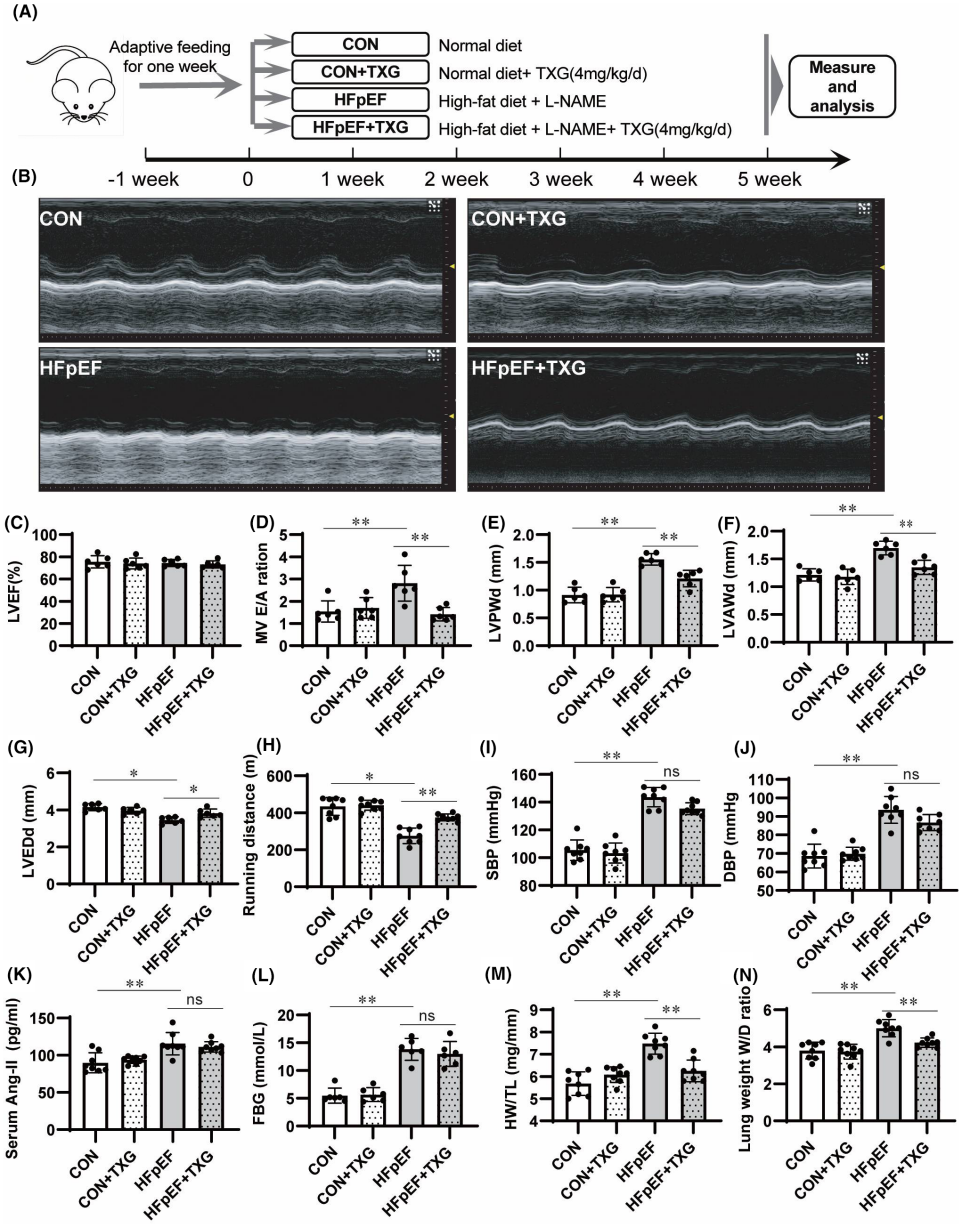
TXG ameliorates cardiac dysfunction of HFD + L‐NAME‐induced Heart failure with preserved ejection fraction (HFpEF) mice. (A) Schematic illustrations for the protocol of high‐fat diet (HFD) and L‐NAME (HFD + L‐NAME) induced HFpEF mice and TXG treatment. (B) Representative left ventricular M‐mode echocardiography in mice fed with normal diet or HFD + L‐NAME with or without TXG treatment. Statistical analysis of cardiac ultrasound parameters (*n* = 6 per group), including left ventricular ejection fraction (LVEF) (C), MV E/A ratio (D), LV end‐diastolic posterior wall thickness (LVPWd) (E), LV end‐diastolic anterior wall thickness (LVAWd) (F), LV end‐diastolic internal diameter (LVEDd) (G) across the different groups. Statistical analysis of running distance (H), SBP (I), DBP (J), Serum Ang‐II (K), fasting blood glucose (FBG) (L), heart weight to tibia length ratio (HW/TL) (M) and lung weight wet/dry ratio (N) in mice feed with normal diet or HFD + L‐NAME with or without TXG. Data are shown as the mean ± SD. Significance: *p < 0.05; **p < 0.01; ns represent no significant difference.

The mouse model of HFD + L‐NAME‐induced HFpEF was established as described previously.^6^, ^7^, ^12^, ^13^ In this study, male C57BL/6N wild‐type mice were divided into four groups after 1 week of adaptive training: Control (CON) group, xanthone treatment (TXG) group, HFpEF model (HFpEF) group and HFpEF model treated with xanthone (HFpEF + TXG) group (Figure 1A). In line with previous findings, the results of echocardiography analysis (Figure 1B) revealed that mice fed with HFD + L‐NAME for 5 weeks exhibited no significant change in LVEF (Figure 1C), accompanied with increases in mitral valve E/A ratio (MV E/A) (Figure 1D), LVPWd (Figure 1E) and LVAWd (Figure 1F), as well as a decrease in LVEDd (Figure 1G), suggesting successful induction of the HFpEF phenotype in the HFD + L‐NAME model. Interestingly, TXG treatment reversed these pathological changes (Figure 1D–G). Moreover, TXG improved the exercise dysfunction in mice with HFD + L‐NAME‐induced HFpEF, showing a significant decrease in exercise endurance compared to that in the CON group (Figure 1H). Additionally, the mice with HFD + L‐NAME‐induced HFpEF also exhibited hypertension (Figure 1I,J), elevated plasma AngII level (Figure 1K), and hyperglycaemia (Figure 1L), which were not significantly reversed by TXG treatment. Furthermore, heart weight/tibia length ratio (HW/TL) (Figure 1M) and lung wet weight/dry weight ratio (Figure 1N) were increased in HFpEF mice manifesting myocardial hypertrophy and pulmonary oedema, which were also improved by TXG treatment. These results suggested that TXG has a therapeutic effect on HFpEF.

### 3.2 TXG ameliorated myocardial hypertrophy and fibrosis in HFD + L‐NAME‐induced HFpEF mice

**FIGURE 2:**
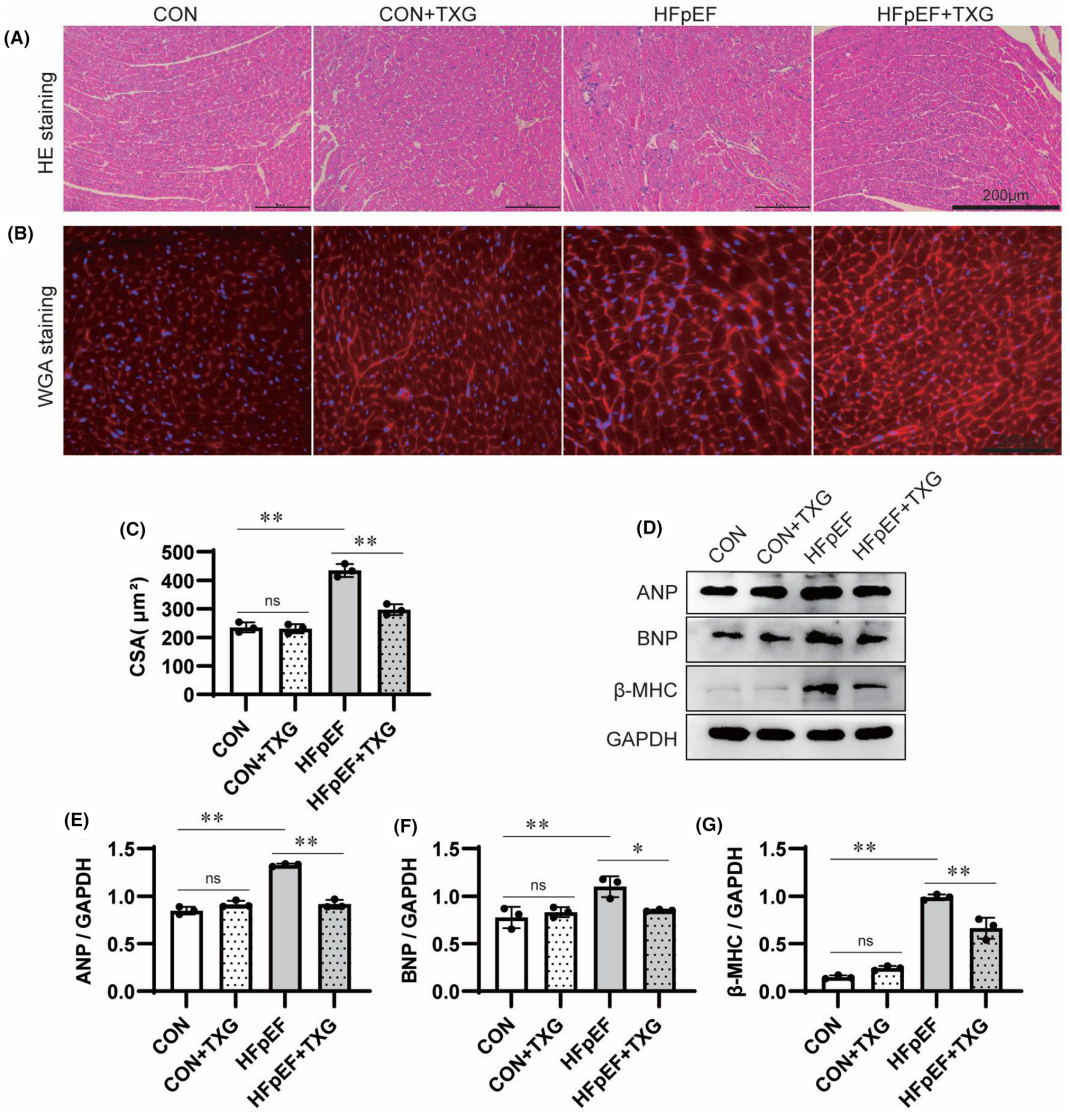
TXG ameliorates cardiac hypertrophy and fibrosis in the HFD + L‐NAME‐induced HFpEF mice. (A) Representative images of HE staining of left ventricular tissue in different groups; scale bars = 200 μm. (B) Representative images of WGA staining of left ventricular tissue in different groups; scale bars = 200 μm. (C) Quantitative analysis of cardiomyocyte diameter in different groups. (D) Western blot analysis of ANP, BNP, and β‐MHC expression in cardiac tissues in different groups. Statistical analysis of protein level of ANP (E), BNP (F) and β‐MHC (G) in cardiac tissues in different groups, GAPDH is used as a loading control. Data are shown as the mean ± SD (*n* = 3). Significance: *p < 0.05; **p < 0.01; ns represent no significant difference.

**FIGURE 3:**
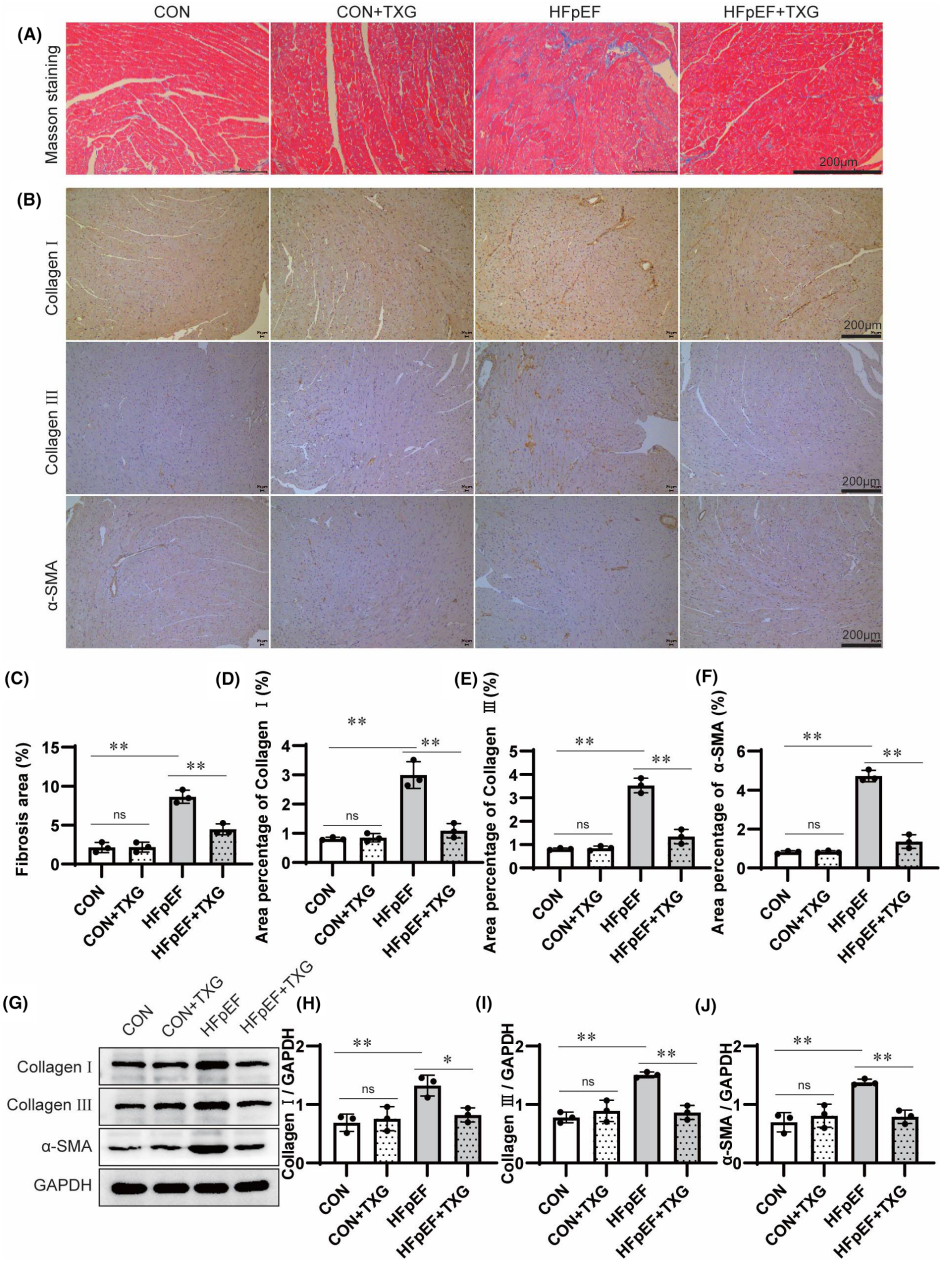
TXG ameliorates cardiac fibrosis in the HFD + L‐NAME‐induced HFpEF mice. (A) Representative images of Masson trichrome staining of left ventricular tissue in different groups; scale bars = 200 μm. (B) Representative images of immunohistochemical staining of collagen I, collagen III and α‐SMA of left ventricular tissue in different groups; scale bars = 200 μm. (C) Quantitative analysis of collagen volume fraction in different groups. Statistical analysis of Collagen I (D), Collagen III (E), α‐SMA (F) area in left ventricular tissue in different groups. (G) Western blot analysis of Collagen I, Collagen III and α‐SMA expression in cardiac tissues in different groups. Statistical analysis of protein level of Collagen I (H), Collagen III (I) and α‐SMA (J) in cardiac tissues in different groups, GAPDH is used as a loading control. Data are shown as the mean ± SD (*n* = 3). Significance: *p < 0.05; **p < 0.01; ns represent no significant difference.

In order to investigate the protective mechanism of TXG against HFpEF, we further explored the effects of TXG on myocardial hypertrophy and fibrosis, which were known as the risk factors of heart failure. HE staining (Figure 2A) and WGA staining (Figure 2B) revealed a significant increase in cardiomyocyte diameter in HFpEF mice compared to that in the controls (Figure 2C), while TXG treatment reversed myocardial hypertrophy (Figure 2A–C). These results supported the echocardiography data. Compared to the CON group, the HFpEF group exhibited a significant increase in heart wall thickness, but such a pathological change was inhibited after TXG treatment. Consistent with the results of cell morphology analysis, elevated expression levels of atrial natriuretic peptide (ANP), BNP and β‐MHC were observed in the HFpEF group compared to those in the CON group. Nevertheless, these abnormal expressions were reduced upon TXG treatment (Figure 3D–G). Additionally, Masson's trichrome staining revealed a significant reduction in enhanced collagen deposition in mice with HFD + L‐NAME‐induced HFpEF after TXG treatment (Figure 3A). Immunohistochemical staining (Figure 3B–F) and Western blotting analysis (Figure 3G–J) demonstrated that TXG treatment attenuated the overexpressions of Collagen I, Collagen III and α‐SMA in HFpEF mice. These data indicated that TXG effectively repairs and reverses cardiac remodelling at both molecular and structural levels in HFpEF mice.

### 3.3 TXG ameliorated cardiomyocyte apoptosis in HFpEF mice

**FIGURE 4:**
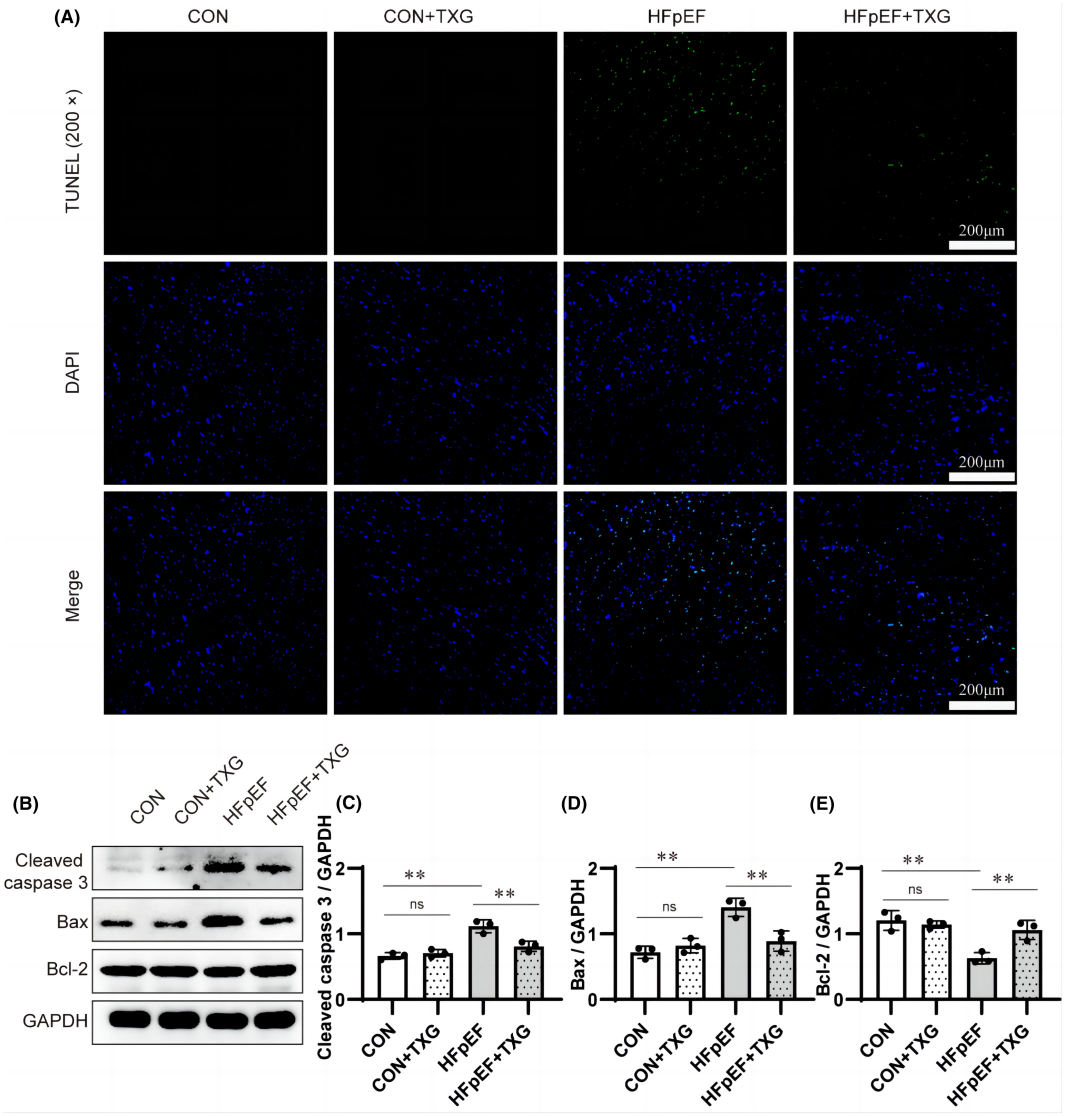
TXG ameliorates cardiomyocyte apoptosis in HFpEF mice. (A) Representative images of TUNEL assay and DAPI staining in different groups. (B) Western blot analysis of Cleaved caspase 3, Bax and Bcl‐2 in cardiac tissues in different groups. Statistical analysis of protein level of Cleaved caspase 3 (C), Bax (D), Bcl‐2 (E) in different groups. GAPDH is used as a loading control. Data are shown as the mean ± SD (*n* = 3). Significance: **p < 0.01; ns represent no significant difference.

Apoptosis has been proved to play an important role in the pathophysiology of HFpEF.^14^, ^15^ In this study, the effect of TXG on cardiomyocyte apoptosis was further investigated. The results indicated that TXG effectively reduced the apoptosis in the myocardial tissue of HFpEF mice. TUNEL assay results showed a significant increase in cardiomyocyte apoptosis in HFpEF mice, which was effectively alleviated after TXG treatment (Figure 4A). Furthermore, the expression level of apoptosis‐related markers such as Cleaved caspase 3, Bax and Bcl‐2 was consistent with TUNEL assay results. Western blotting (Figure 4B) results indicated the up‐regulation of Cleaved caspase 3 (Figure 4C) and Bax (Figure 4D), together with the down‐regulation of Bcl‐2 (Figure 4E), in the myocardial tissue of HFpEF mice, while these trends were reversed subsequent to TXG treatment (Figure 4B–E). These findings demonstrated that TXG can effectively relieve the cardiomyocyte apoptosis in HFpEF mice.

### 3.4 TXG ameliorated cardiac remodelling through activating IRE1α phosphorylation in HFpEF mice

**FIGURE 5:**
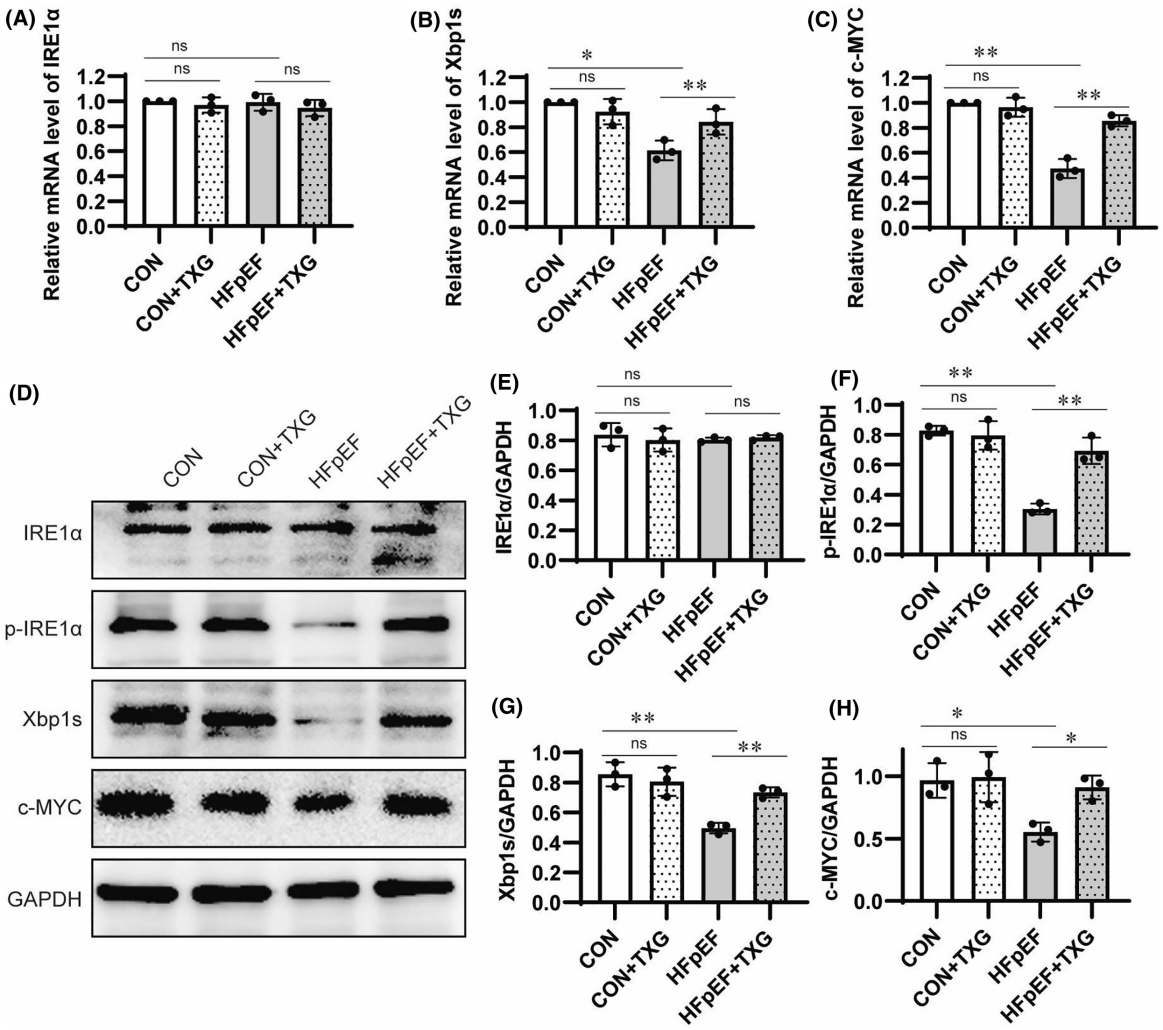
TXG activates IRE1α/Xbp1s signalling pathway in HFpEF mice. Real‐time PCR analysis of IRE1α (A), Xbp1s (B) and c‐MYC (C) expression in cardiac tissues in different groups. (D) Western blot analysis of IRE1α, p‐IRE1α, Xbp1s and c‐MYC in cardiac tissues in different groups. Statistical analysis of protein level of IRE1α (E), p‐IRE1α (F), Xbp1s (G) and c‐MYC (H) in different groups. GAPDH is used as a loading control. Data are shown as the mean ± SD (*n* = 3). Significance: *p < 0.05; **p < 0.01; ns represent no significant difference.

Phosphorylated IRE1α (p‐IRE1α) has been identified as a crucial factor in the HFD + L‐NAME‐induced HFpEF model.^6^, ^16^ S‐nitrosylation can stimulate IRE1α degradation to cause Xbp1 splicing defects, thus triggering the clinical symptoms associated with HFpEF.^6^ To determine whether IRE1α plays a role in the alleviation of HFpEF by TXG, we further explored the effect of TXG on the IRE1α/Xbp1s signalling pathway in the case of HFpEF. RT‐PCR analysis demonstrated that TXG failed to increase the transcriptional level of IRE1α (Figure 5A), whereas its downstream target gene Xbp1 (Figure 5B) and apoptosis regulatory factor c‐MYC^17^ (Figure 5C) were increased in HFpEF. Furthermore, Western blotting results presented no changes in IRE1α protein expression (Figure 5D,E), which was consistent with the results at the transcriptional level. However, significantly decreased p‐IRE1α level was detected (Figure 5D,F) in HFpEF. Furthermore, down‐regulation of Xbp1 (Figure 5D,G) and c‐MYC (Figure 5D,H) at the protein expression level was observed in HFpEF mice. Such down‐regulation was reversed by TXG treatment (Figure 5B–D,F–H). Based on these findings, p‐IRE1α plays a critical role in cardiomyocyte apoptosis during HFpEF progression, and TXG effectively alleviates cardiomyocyte apoptosis by restoring p‐IRE1α expression.

### 3.5 TXG ameliorated AngII‐induced H9c2 cell apoptosis in an IRE1α/Xbp1s signalling pathway‐dependent manner

**FIGURE 6:**
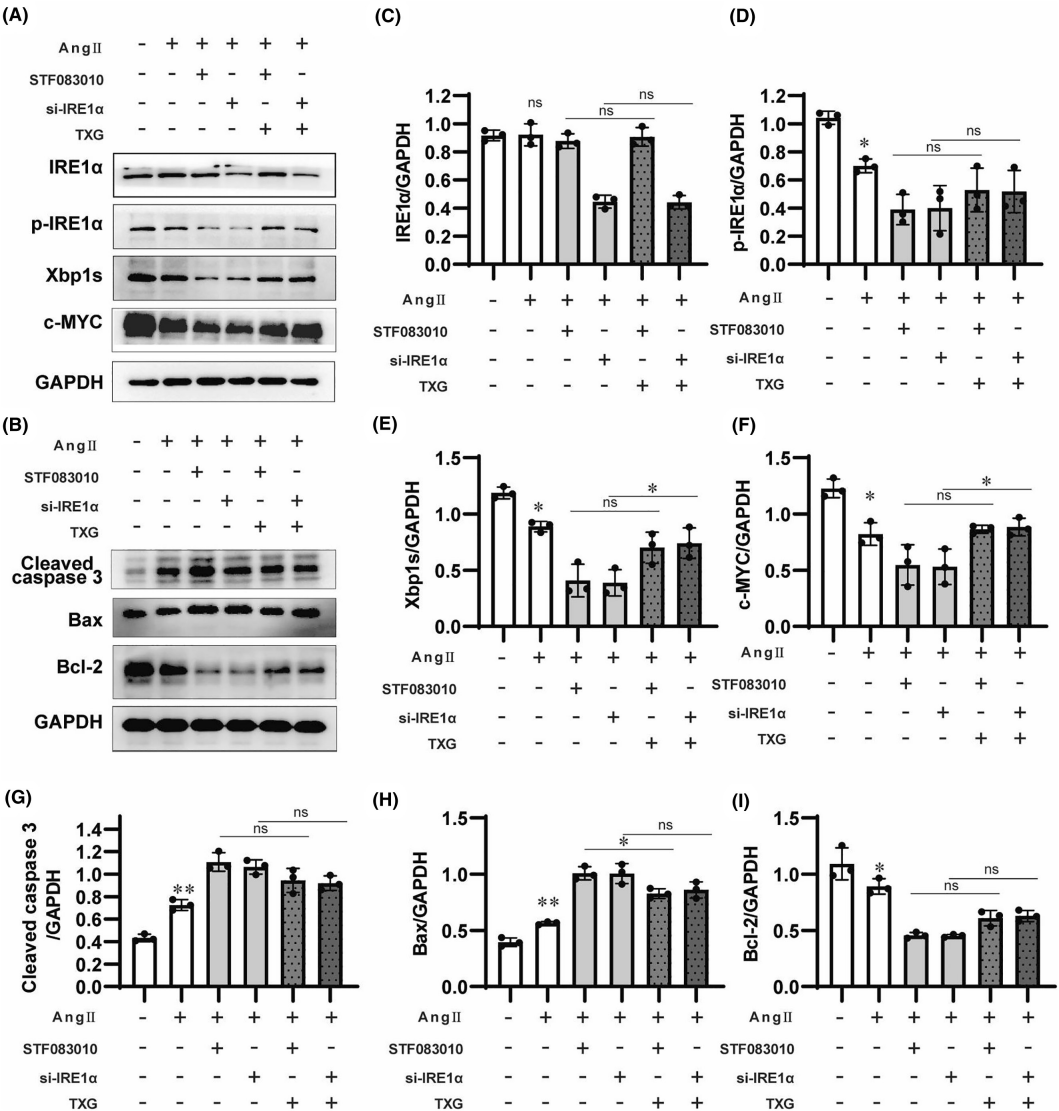
TXG ameliorates AngII‐induced cardiac apoptosis in an IRE1α/Xbp1s signalling‐dependent manner in H9c2 cells. (A) Western blot analysis of IRE1α, p‐IRE1α, Xbp1s and c‐MYC in H9c2 cells treated with STF083010 or siRNA‐IRE1α with/without TXG. (B) Western blot analysis of Cleaved caspase 3, Bax, Bcl‐2 in H9c2 cells treated with STF083010 or siRNA‐IRE1α with/without TXG. Statistical analysis of protein level of IRE1α (C), p‐IRE1α (D), Xbp1s (E), c‐MYC (F), Cleaved caspase 3 (G), Bax (H), Bcl‐2 (I) in different groups. GAPDH is used as a loading control. Data are shown as the mean ± SD (*n* = 3). Significance: *p < 0.05; **p < 0.01; ns represent no significant difference.

The plasma AngII was increased in mice with HFD + L‐NAME‐induced HFpEF in this study, which has been testified to induce cardiomyocyte apoptosis.^18^, ^19^ Therefore, the AngII‐induced cardiomyocyte apoptosis model was constructed in this study. To investigate whether IRE1α plays a crucial role in cardiomyocyte apoptosis, the IRE1α inhibitor STF083010 and IRE1α siRNA (si‐IRE1α) were applied to mediate p‐IRE1α expression in H9c2 cells. STF083010 and si‐IRE1α exerted their effects on p‐IRE1α expression through distinct mechanisms. Specifically, STF083010 selectively inhibited IRE1α ribonuclease activity to lower the level of p‐IRE1α‐mediated Xbp1s. Consistent with previous studies,^20^ STF083010 had no influence on the total protein expression of IRE1α but decreased the p‐IRE1α level (Figure 6A,C,D), thereby decreasing the downstream targets Xbp1s and c‐MYC (Figure 6A,E,F). On the other hand, si‐IRE1α reduced the protein expression of IRE1α that also led to reductions in p‐IRE1α and its downstream targets (Figure 6A,C–F). In this study, an AngII‐induced apoptosis model was established in H9c2 cells. The results demonstrated a significant up‐regulation of Cleaved caspase 3 and Bax, along with concurrent down‐regulation of Bcl‐2, which were induced by AngII (Figure 6B,G–I). The inhibition of IRE1α resulted in increased apoptosis, significant increases in Cleaved caspase 3 and Bax expressions, and a decrease in Bcl‐2 (Figure 6B,G–I). The aforementioned findings were consistent with the observations in HFpEF mice, while decreased p‐IRE1α promoted cardiomyocyte apoptosis. Interestingly, TXG treatment enhanced p‐IRE1a expression, consequently resulting in the reduction of Cleaved caspase 3 and Bax, together with the increment of Bcl‐2 expression in HFpEF mice. On the contrary, TXG could not reverse the AngII‐induced increases in Cleaved caspase 3 and Bax, and decrease in Bcl‐2 expressions in H9c2 cells treated with STF083010 or si‐IRE1α (Figure 6B,G,H). This could be potentially attributed to the fact that STF083010 and si‐IRE1α effectively diminish p‐IRE1α expression, during which TXG cannot effectively restore the p‐IRE1 level (Figure 6D). These findings suggest that the IRE1α/Xbp1s signalling pathway is a critical regulator for TXG in ameliorating cardiomyocyte apoptosis in the case of HFpEF.

## 4 DISCUSSION

**FIGURE 7:**
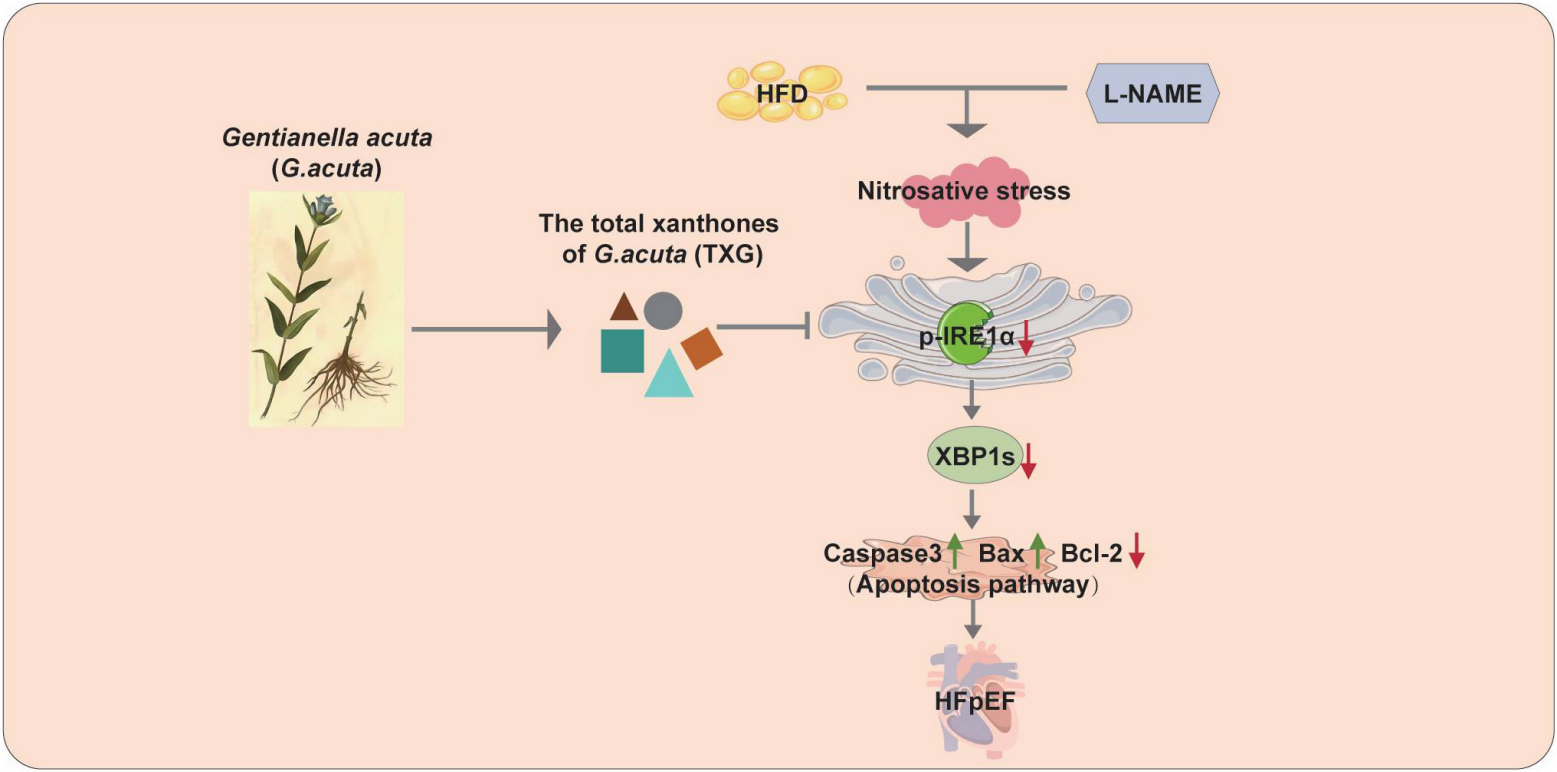
Schematic of TXG ameliorates cardiac remodelling in HFD + L‐NAME‐induced HFpEF mice. The total xanthones extracted from *Gentianella acuta* (TXG) activates IRE1α/Xbp1s signalling to reduce cardiac hypertrophy, fibrosis and apoptosis‐related genes, leading to ameliorate HFpEF.

This study aims to investigate the therapeutic potential of TXG in improving myocardial remodelling and apoptosis in mice with HFpEF induced by HFD and L‐NAME, so as to provide an effective treatment strategy for HFpEF. Our results showed that TXG significantly reduced biomarkers related to myocardial hypertrophy, fibrosis, and apoptosis in mice with HFD + L‐NAME‐induced HFpEF via the IRE1α/Xbp1s signalling pathway (Figure 7). The findings demonstrate that TXG significantly ameliorates cardiac dysfunction in HFpEF mice, suggesting the therapeutic potential of TXG for HFpEF.

Studies have reported that traditional Chinese medicine can improve the cardiac structure and function of patients with chronic heart failure, with good safety.^21^ Therefore, it boasts great potential in the treatment of cardiovascular diseases. For instance, YiQi Huoxue Recipe can reduce the infarct area of cardiac tissue, inhibit cardiomyocyte apoptosis, regulate apoptosis pathway, increase the Bcl‐2/Bax ratio, and reduce Caspase‐3 expression, thus protecting patients with heart failure.^22^ Additionally, oral administration of Xiao‐Qing‐Long Tang (XQLT) can alleviate hypertension, reduce cardiac inflammation and mitigate compensatory hypertrophy via regulating gut microbiota in HFpEF rats.^23^ Our laboratory has been focusing on the therapeutic effect of *G. acuta*, an annual herb of the Gentianaceae family, on cardiac diseases.^9^, ^10^ Previous studies have demonstrated that *G. acuta* improves cardiac remodelling through the Notch and PI3K/Akt/FOXO1/3 signalling pathways.^9^ In addition, *G. acuta* inhibits cardiac fibrosis by suppressing the NF‐κB pathway in isoproterenol‐induced rats, thereby exerting its protective effects.^24^ Xanthones extracted from *G. acuta* have been reported to be effective in reducing myocardial hypertrophy and ameliorating myocardial infarction by inhibiting the inflammatory pathway.^11^, ^25^ Previous studies have also manifested that *G. acuta* prevents myocardial fibrosis via repressing the TGF‐β1/Smads signalling pathway.^10^ Some studies revealed that *G. acuta* effectively prevented ISO‐mediated abnormal proliferation and deformation through suppressing the NOX4/ROS signalling pathway.^11^ In this study, the medicinal application of *G. acuta* was further explored, and it was discovered that TXG significantly improved the signs and histological changes of HFpEF.

About half of the patients with heart failure exhibit cardiac function with preserved normal ejection fraction, which is known as HFpEF.^26^ HFpEF sufferers typically exhibit normal LVEF (≥50%), but already present with severe cardiac structural abnormalities and/or dysfunction, as well as heart failure symptoms involving dyspnea and exercise tolerance decline.^1^ HFpEF patients exhibit severe myocardial hypertrophy and fibrosis, impairing the cardiac elasticity and leading to diastolic dysfunction, so they represent the core pathological features and pathogenesis of HFpEF. As the disease progresses, these abnormalities result in the failure to meet the increasing demand for cardiac output in organisms during physical activity.^27^ In this study, HFD and L‐NAME were successfully applied to induce the pathological process of HFpEF in mice.^6^ Consistent with the findings of a previous study, the mice exhibited the characteristics of HFpEF after 5 weeks of HFD and L‐NAME induction, including no change in LVEF, increased *E*/*A* ratio, raised LVPWd, enlarged LVAWd, decreased LVEDd, significantly reduced exercise capacity, and markedly elevated lung wet/dry weight ratio, along with notable myocardial hypertrophy and fibrosis. Interestingly, TXG effectively ameliorated the aforementioned characteristics in mouse and cell models, while the myocardial hypertrophy‐related proteins (ANP, BNP and β‐MHC) and myocardial fibrosis‐associated proteins (α‐SMA, Collagen I and Collagen III) were reversed to normal levels. These results imply that TXG holds promise as a potential therapeutic agent for HFpEF.

Currently, there are few effective treatments for HFpEF, largely because the underlying pathogenic and molecular mechanisms have not been fully elucidated. Some studies have revealed that endoplasmic reticulum oxidative stress plays an important role in the mouse model of L‐NAME + HFD induced HFpEF. Xbp1s, a crucial regulator of endoplasmic reticulum oxidative stress, is associated with p‐IRE1α decrease caused by S‐nitrosylation of IRE1α.^28^ Therefore, a number of studies have proposed that drug interventions potentially reverse the pathological characteristics of HFpEF by facilitating the up‐regulation of Xbp1s.^28^ In this study, TXG was utilized to treat HFpEF mice and H9c2 cells. It was uncovered that TXG could significantly increase the phosphorylation level of IRE1α, consequently reducing the Xbp1s level and simultaneously improving the markers in relation to myocardial hypertrophy and fibrosis. Therefore, the IRE1α inhibitor (STF083010) and siRNA were used to interfere with the function or expression of IRE1α, in order to further explore whether IRE1α plays a key role in TXG‐mediated HFpEF alleviation. STF083010 is unable to alter the total protein expression of IRE1α, but reduces the level of p‐IRE1α by inhibiting nuclease activity, which is consistent with previous study results.^20^ The inhibitor STF083010 and siRNA‐IRE1α impede the phosphorylation process of IRE1α, and TXG treatment fails to effectively restore the p‐IRE1α level. Consequently, as for AngII‐induced cardiomyocyte apoptosis, TXG treatment combined with IRE1α inhibitor (STF083010) or siRNA cannot preferably mitigate the changes in the expressions of Cleaved caspase 3, Bax and Bcl‐2. Hence, our results demonstrated that TXG exerts protective effects through the IRE1α signalling pathway under HFpEF. As for the limitation of this study, no cell lines or mouse models with IRE1α knockout were created for verification. IRE1α performs a dual role in cell survival and death.

The IRE1α is a key protein in ER stress signal transduction. Some studies suggest that ER stress contributes to myocyte apoptosis and heart failure.^29^, ^30^, ^31^ The ER stress signal pathways are robust activated in a preclinical model of pressure overload‐induced HF with reduced ejection fraction (HFrEF) induced by severe transverse aortic constriction.^32^ In addition, IRE1α/ASK1/JNK pathway and IRE1α/JNK/Beclin‐1pathway plays critical roles in Doxorubicin‐induced cardiac inflammation, apoptosis, and autophagy.^33^ However, the IRE1α also is a key protein in HFpEF progress, but, as a protective fact. IRE1α phosphorylation levels were also down‐regulated exclusively in human HFpEF myocardium, but not in HFrEF. The Nitrosative Stress dysregulation of IRE1α‐Xbp1s as a crucial mechanism of cardiomyocyte dysfunction in HFpEF, leaded to cardiomyocyte lipotoxicity and inflammation,^7^, ^16^ and improve the IRE1α phosphorylation levels ameliorated the HFpEF phenotype in mice mode.^6^ To study the performance of TXG in relieving HFpEF, particularly its relation to IRE1α dependence, TXG was deemed more appropriate for the cell model featured by partial knockout of IRE1α.

Despite the fairly limited investigations into the mechanism of cardiomyocyte apoptosis in HFpEF, most studies on HFpEF have consistently demonstrated that cardiomyocyte apoptosis is a pivotal player in exacerbating heart failure and holds significant pathophysiological implications for the onset and progression of this disease.^34^ In the HFD + L‐NAME‐induced HFpEF mouse model, a significant increase in cardiomyocyte apoptosis accompanied by up‐regulation of apoptosis‐related biomarkers was observed, indicating the occurrence of cardiomyocyte apoptosis in HFpEF. This phenomenon was effectively mitigated by TXG treatment. To further validate this finding, the apoptosis in H9c2 cells was induced using AngII. In addition to the application of typical apoptotic molecules such as Cleaved caspase 3, Bax and Bcl‐2, the effect of TXG on c‐MYC expression was also explored. As a transcription factor, c‐MYC plays a crucial role in cell proliferation, growth, and apoptosis. Some studies have reported that c‐MYC is closely related to the development of cardiac diseases, as it regulates the expression of a variety of genes associated with cell cycle, metabolism and apoptosis. Moreover, it has been unveiled that the overexpression of c‐MYC is associated with cardiomyocyte proliferation and cardiac remodelling. However, c‐MYC is also involved in cardiomyocyte apoptosis and exerts a dual effect on cardiac stress response.^35^ Besides, c‐MYC may promote apoptosis by activating a series of downstream target genes such as Bax, Bcl‐2 family proteins and caspase family proteins.^6^, ^36^ In addition, c‐MYC is one of the important mediators for promoting apoptotic signal transduction, which can initiate programmed death in response to oxidative stress, DNA damage and other signals causing myocardial damage.^37^, ^38^ Relevant studies have manifested that the activity of the IRE1α/Xbp1s signalling pathway is crucial for c‐MYC signalling, and Xbp1s is essential for optimal c‐MYC mRNA and protein expression.^17^ Both Bax and Bcl‐2 serve as transcription targets and mediators in c‐MYC‐induced apoptosis.^39^, ^40^ In this study, the expressions of Xbp1s, C‐MYC and apoptotic markers after IRE1α inhibitor or siRNA treatment were confirmed by virtue of cell experiments, and these expressions showed a highly consistent trend. These results suggest the important role of the IRE1α/Xbp1/c‐MYC signalling pathway in the action mechanism of TXG.

## 5 CONCLUSION

In conclusion, TXG is proven to activate the IRE1α/Xbp1s signalling pathway to mediate myocardial hypertrophy, fibrosis and apoptosis‐related proteins, thereby improving cardiac function in HFpEF.

## AUTHOR CONTRIBUTIONS

**Linna Zhao:** Data curation (lead); methodology (equal); writing – original draft (equal). **Yiping Qin:** Data curation (equal); investigation (equal); writing – original draft (equal). **Yangong Liu:** Investigation (equal); project administration (equal). **Liping An:** Investigation (equal); supervision (equal); visualization (equal). **Weizhe Liu:** Investigation (equal); supervision (equal); visualization (equal). **Chuang Zhang:** Data curation (supporting); visualization (equal). **Qiuhang Song:** Validation (equal); visualization (equal). **Cheng Dai:** Supervision (equal). **Juanjuan Zhang:** Funding acquisition (equal); validation (equal); writing – review and editing (lead). **Aiying Li:** Conceptualization (equal); funding acquisition (equal); project administration (lead); supervision (equal).

## CONFLICT OF INTEREST STATEMENT

The authors declare that there are no known competing financial interests or personal relationships that could have appeared to influence the work reported in this paper.

## Supporting information


**Figure S1.** HPLC analysis of total xanthones extracted from *Gentianella acuta* and Standard Xanthone Samples. (A) High‐performance liquid chromatography (HPLC) Analysis of standard xanthone samples, including mangiferin (A), norswertianolin (B), swertianolin (C), demethylbellidifolin (D), and bellidifolin (E); (B) HPLC Analysis of Total Xanthones Extracted from *Gentianella acuta*.

## Data Availability

The data are available from the corresponding author on reasonable request.
