# Supplementary figures and images for: The total xanthones extracted from Gentianella acuta alleviates HFpEF by activating the IRE1α/Xbp1s pathway
Source: J Cell Mol Med. 2024 Jun 7;28(11):e18466. doi: 10.1111/jcmm.18466 (PMC11157675; doi:10.1111/jcmm.18466)

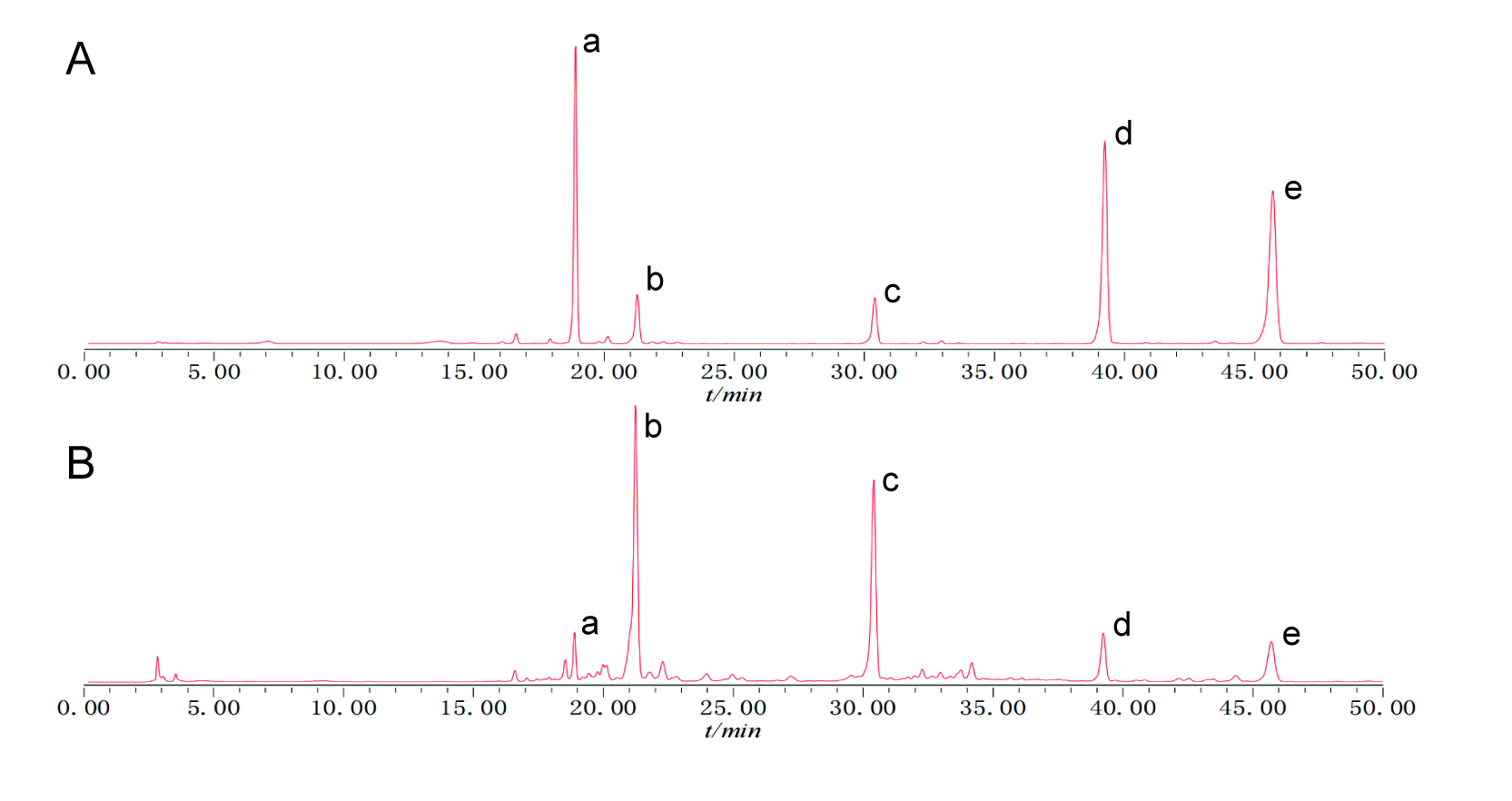

Supplement: Supplementary file 1 — Figure S1. HPLC analysis of total xanthones extracted from Gentianella acuta and Standard Xanthone Samples. (A) High‐performance liquid chromatography (HPLC) Analysis of standard xanthone samples, including mangiferin (A), norswertianolin (B), swertianolin (C), demethylbellidifolin (D), and bellidifolin (E); (B) HPLC Analysis of Total Xanthones Extracted from Gentianella acuta. [file JCMM-28-e18466-s001.tif]
